# Diagnostic performance of a single and duplicate Kato-Katz, Mini-FLOTAC, FECPAK^G2^ and qPCR for the detection and quantification of soil-transmitted helminths in three endemic countries

**DOI:** 10.1371/journal.pntd.0007446

**Published:** 2019-08-01

**Authors:** Piet Cools, Johnny Vlaminck, Marco Albonico, Shaali Ame, Mio Ayana, Barrios Perez José Antonio, Giuseppe Cringoli, Daniel Dana, Jennifer Keiser, Maria P. Maurelli, Catalina Maya, Leonardo F. Matoso, Antonio Montresor, Zeleke Mekonnen, Greg Mirams, Rodrigo Corrêa-Oliveira, Simone A. Pinto, Laura Rinaldi, Somphou Sayasone, Eurion Thomas, Jaco J. Verweij, Jozef Vercruysse, Bruno Levecke

**Affiliations:** 1 Department of Virology, Parasitology and Immunology, Ghent University, Merelbeke, Belgium; 2 Center for Tropical Diseases, Sacro Cuore Don Calabria Hospital, Negrar, Italy; 3 Department of Life Sciences and Systems Biology, University of Turin, Italy; 4 Public Health Laboratory-Ivo de Carneri, Chake Chake, United Republic of Tanzania; 5 Jimma University Institute of Health, Jimma University, Jimma, Ethiopia; 6 Engineering Institute of National Autonomous University of Mexico, Mexico City, Mexico; 7 Department of Veterinary Medicine and Animal Production, University of Naples Federico II, Naples, Italy; 8 Department of Medical Parasitology and Infection Biology, Swiss Tropical and Public Health Institute, Basel, Switzerland; 9 University of Basel, Basel, Switzerland; 10 Laboratory of Molecular and Cellular Immunology, Research Center René Rachou—FIOCRUZ, Belo Horizonte, Brazil; 11 Department of Control of Neglected Tropical Diseases, World Health Organization, Geneva, Switzerland; 12 Techion Group Ltd, Dunedin, New Zealand; 13 Lao Tropical and Public Health Institute, Ministry of Health, Vientiane, Lao People’s Democratic Republic; 14 Techion Group Ltd, Aberystwyth, United Kingdom; 15 Laboratory for Medical Microbiology and Immunology, Elisabeth-Tweesteden Hospital, Tilburg, The Netherlands; Emory University, UNITED STATES

## Abstract

**Background:**

Because the success of deworming programs targeting soil-transmitted helminths (STHs) is evaluated through the periodically assessment of prevalence and infection intensities, the use of the correct diagnostic method is of utmost importance. The STH community has recently published for each phase of a deworming program the minimal criteria that a potential diagnostic method needs to meet, the so-called target product profiles (TPPs).

**Methodology:**

We compared the diagnostic performance of a single Kato-Katz (reference method) with that of other microscopy-based methods (duplicate Kato-Katz, Mini-FLOTAC and FECPAK^G2^) and one DNA-based method (qPCR) for the detection and quantification of STH infections in three drug efficacy trials in Ethiopia, Lao PDR, and Tanzania. Furthermore, we evaluated a selection of minimal diagnostic criteria of the TPPs.

**Principal findings:**

All diagnostic methods showed a clinical sensitivity of ≥90% for all STH infections of moderate-to-heavy intensities. For infections of very low intensity, only qPCR resulted in a sensitivity that was superior to a single Kato-Katz for all STHs. Compared to the reference method, both Mini-FLOTAC and FECPAK^G2^ resulted in significantly lower fecal egg counts for some STHs, leading to a substantial underestimation of the infection intensity. For qPCR, there was a positive significant correlation between the egg counts of a single Kato-Katz and the DNA concentration.

**Conclusions/Significance:**

Our results indicate that the diagnostic performance of a single Kato-Katz is underestimated by the community and that diagnostic specific thresholds to classify intensity of infection are warranted for Mini-FLOTAC, FECPAK^G2^ and qPCR. When we strictly apply the TPPs, Kato-Katz is the only microscopy-based method that meets the minimal diagnostic criteria for application in the planning, monitoring and evaluation phase of an STH program. qPCR is the only method that could be considered in the phase that aims to seek confirmation for cessation of program.

**Trial registration:**

ClinicalTrials.gov NCT03465488

## Author summary

To control the burden caused by intestinal worms, the World Health Organization recommends large-scale deworming programs where anti-worm drugs are administered to at-risk populations. The decision to scale down drug distribution is based on the periodically assessment of prevalence and intensity of infections using a standard diagnostic method. Today, the scientific community strongly doubts whether this method can be used throughout the program. This is in particular when it fails to detect infections of low intensity, and hence may result in prematurely stopping the distribution of drugs. We compared the diagnostic performance of alternative diagnostic methods in three drug efficacy trials in two African and one Asian country. The diagnostic methods were based on demonstration of worm eggs or worm DNA in stool. We also checked the results with minimal diagnostic criteria which have been recently been proposed by the scientific community. Our results indicate that of all diagnostic methods based on demonstration of worm eggs, only the current standard method fulfills the diagnostic criteria for planning, monitoring and evaluation phases of deworming program. Furthermore, we showed that DNA-based methods could be considered in the phase that aims to seek confirmation for cessation of the deworming program.

## Introduction

Soil-transmitted helminths (STHs) are a group of intestinal parasitic worms that excrete eggs through feces, which contaminate soil in areas where sanitation is poor and infect the human host orally or through skin contact [1]. The main STH species are the giant roundworm (*Ascaris lumbricoides*), the whipworm (*Trichuris trichiura*), and the two hookworms (*Necator americanus* and *Ancylostoma duodenale*) [2]. They affect one fifth of the world population, mainly in sub-tropical and tropical regions, and are responsible for more than three million disability-adjusted life years [1, 3].

The current global strategy set by the World Health Organization (WHO) is to control the morbidity from STH infections in at-risk populations (i.e. pre-school-aged children (pre-SAC), school-aged children (SAC), women of reproductive age and adult groups particularly exposed to STHs, such as tea-pickers and miners), with the ultimate goal to eliminate STH infections as a public health problem [4]. For operational purposes, STH infections are defined as a public health problem if the prevalence of moderate-to-heavy intensity of any STH infection is more than 1% in the at-risk populations [5]. To accomplish this goal, endemic countries implement preventive chemotherapy (PC) programs, during which anthelmintic drugs (albendazole (ALB) or mebendazole (MEB)) are administered to all individuals of a target population, regardless of their individual infection status. Boosted by the London Declaration on NTDs, drug donations have increased from 30% to a current global PC coverage of 63% in pre-SAC and SAC [6]. This successful and ongoing scaling-up of STH PC programs has substantially reduced the morbidity due to STH infections [7–9].

Diagnostics provide essential information for STH control programs, as they inform on the progress made in reducing both prevalence and intensity of infections, and indicate when scaling down in frequency in PC is justified. Currently, the diagnosis of STHs relies on the microscopic demonstration of eggs in stool and the Kato-Katz is the most widely used method. Although Kato-Katz is cheap and simple, it often fails to detect infections of low intensity [10]. Another chief limitation of the Kato-Katz method is the different clearing times across the different STH eggs. Hookworm eggs rapidly disappear in cleared slides, resulting in false negative test results if the interval between preparation and examination of the slides is too long (≥60 min). These differences in clearing time not only impede simultaneous detection of all STHs, it also impedes quality control of the egg counts across the different STHs.

To pave the way in addressing the need for more sensitive diagnostic methods [11], a group of key STH diagnostic experts recently published a framework in which they define four specific intended uses, named ‘use-cases’ [11]. Use-case #1 concerns whether or not to start PC in a certain population and at which frequency (annually or bi-annually). Use-case #2 applies to STH programs that have initiated PC and aim to assess progress against program goals. Use-case #3 relates to programs that aim to interrupt transmission and seek confirmation to stop PC whereas use-case #4 applies to programs that seek to verify sustained interruption of transmission. In addition, they defined for each use-case the minimal and ideal (optimistic) criteria that a diagnostic method should meet (referred to as *target product profiles* (TPPs)). An overview of use-case #1 to #3 with corresponding key minimal and optimistic criteria of TPP #1 to #3 is provided in Table 1, for use-case #4, the TPP still needs to be defined.

**Table 1:** Three use-cases and their accompanying target product profiles for diagnostics in soil-transmitted helminth control programs. This Table is based on a framework described by Lim and colleagues [11]. The Table provides a brief overview of the target product profiles (TPPs) for three out of the 4 described use-cases (#1–3) defined for soil-transmitted helminth (STH) preventive chemotherapy (PC) programs.

	**Use-case #1**	**TPP #1**
Determine STH transmission and identify the type of PC		***Clinical sensitivity*** Minimal criteria: ≥95% for all three STH species compared to a single Kato-Katz for moderate-to-heavy intensity infections, equally sensitive for all three STH species compared to a single Kato-Katz for low infection intensities Optimal criteria: ≥95% for *Schistosoma mansoni* compared to a single Kato-Katz for moderate-to-heavy intensity infections ***Technology*** Minimal criteria: optical microscopy Optimal criteria: automated microscope with autonomous STH egg differentiation and counting, data export for external quality assurance
	**Use-case #2**	**TPP #2**
	Assess progress against program goals	Identical as TPP #1
	**Use-case #3**	**TPP #3**
	Confirm decision to suspend PC intervention and transition to surveillance	***Clinical sensitivity*** Minimal criteria: higher than a single Kato-Katz ***Sample*** Minimal criteria: easily collected biospecimens such as stool, urine, blood or saliva Optimal criteria: non-stool specimens, assay can test pooled specimens

In the present study, we aimed to evaluate the diagnostic performance of five different diagnostic methods for the detection and quantification of STHs in stool. The diagnostic methods included four microscopy-based methods (single and duplicate Kato-Katz, Mini-FLOTAC and FECPAK^G2^) and one DNA-based method (qPCR). These methods were based on their recommendation in a variety of WHO manuals (Kato-Katz and Mini-FLOTAC) [12, 13], their higher sensitivity compared to the currently recommended Kato-Katz (Mini-FLOTAC and qPCR) [10, 14], the potential opportunities for automated egg counting and quality control (FECPAK^G2^) [15], and the ability to simultaneously diagnose diseases other than STH infections (qPCR) [14]. Based on the obtained results, we verified to which extent these methods met any of the aforementioned TPPs, and hence whether they could be implemented in any of the intended use-cases of an STH control program.

## Methodology

### Ethics

The trial was registered at ClinicalTrials.gov (NCT03465488). The study protocol was reviewed and approved by the Institutional Review Board (IRB) of the Faculty of Medicine and Health Sciences of Ghent University, Belgium (EC UZG 2016/0266). The trial protocol was subsequently reviewed and approved by the IRBs associated with each trial site (IRB of Centro de Pesquisas René Rachou, Belo Horizonte, Brazil: 2.037.205; Ethical Review Board of Jimma University, Jimma, Ethiopia: RPGC/547/2016; National Ethics Committee for Health Research (NECHR), Vientiane, Lao PDR: 018/NECHR; Zanzibar Medical Research and Ethics Committee, United Republic of Tanzania: ZAMREC/0002/February/2015). Parent(s) or guardians of participants were asked to sign an informed consent document indicating that they understood the purpose of and procedures required for the study and that they were willing to let their child participate. If the child was ≥5 years, he or she had to orally assent in order to participate in the study. Participants of ≥12 years of age were only included if they signed an informed consent document indicating that they understood the purpose of the study and procedures required for the study and were willing to participate.

### Study design and population

Starworms (an acronym of Stop Anthelmintic Resistant Worms) is a Bill & Melinda Gates Foundation funded project with the overall aim to strengthen the monitoring and surveillance of the drug efficacy and anthelmintic resistance in programs aimed at controlling STHs [16]. The present study is part of work package 1 of the Starworms study and was designed as a series of drug efficacy trials in four STH endemic countries (Brazil, Ethiopia, Lao PDR and Tanzania). The selection of these sites was based on their experience in assessing drug efficacy [17, 18], evaluating the performance of diagnostic methods, the availability of well-equipped diagnostic facilities and skilled personnel, and PC history [19]. Note that the initial study protocol included a study site in Brazil. However, due to the low number of cases on which not all diagnostic methods were performed, the site was excluded from this report.

The trials were designed to assess the equivalence in efficacy of a single oral dose of 400 mg ALB against STH infections in SAC measured by a variety of microscopy-based and DNA-based diagnostic methods. The study focused on SAC (age 5–14) since they are the major target of PC programs, and they usually represent the group with highest worm burdens for *A*. *lumbricoides* and *T*. *trichiura* [2]. The protocol has previously been described in detail [19]. Briefly, at the start of each trial, schools were visited by the local principal investigator and a team of field officers, who explained the planned trial and sampling method to the parents, teachers and the children. At baseline, SAC were asked to provide a fresh stool sample. All children that met all inclusion criteria and none of the exclusion criteria (see S1 Info) were enrolled in the study. They were treated with a single oral dose of 400 mg ALB under supervision. The ALB used in the different studies originated from the same production batch (GlaxoSmithKline, Batch N°: 335726) and was provided by WHO. All collected stool samples were processed to determine the fecal egg counts (FECs; expressed in eggs per gram of stool (EPG)) for each STH using Kato-Katz (single and duplicate), Mini-FLOTAC and FECPAK^G2^. As the latter method was not finalized on the day of sample collection (see section *FECPAK*^*G2*^), results of the FECPAK^G2^ were not used to select individuals for inclusion in follow-up. A subset of the baseline samples, i.e. samples with a FEC of at least 150 EPG for at least one of the STHs measured by either duplicate Kato-Katz or Mini-FLOTAC, were preserved in ethanol (96%) and stored at room temperature for further molecular analysis. Fourteen to twenty-one days after drug administration, a second stool sample was collected from all the children that were excreting eggs of any STH at baseline based on duplicate Kato-Katz and Mini-FLOTAC. These stool samples were examined by Kato-Katz (single and duplicate), Mini-FLOTAC and FECPAK^G2^. All follow-up samples were preserved for further molecular analysis.

### Stool-based microscopic methods

Upon arrival in the laboratory, stool samples were well homogenized and subjected to microscopic examination by means of Kato-Katz (single and duplicate), Mini-FLOTAC and FECPAK^G2^. The standard operating procedures for each of these methods are described elsewhere [19, 20]. In the following paragraphs, the key steps for each of the methods and the actions that were undertaken to secure quality of the egg counting are only briefly described.

#### Single and duplicate Kato-Katz

Two Kato-Katz slides (A and B slide) were prepared per sample and all slides were examined for the presence of STH eggs within 30–60 min following preparation. The number of *A*. *lumbricoides*, *T*. *trichiura* and hookworm eggs were counted and recorded per slide. For the duplicate Kato-Katz, the counts of *A*. *lumbricoides*, *T*. *trichiura* and hookworm eggs of the two slides were added. Slide A was always used to calculate the diagnostic parameters of a single Kato-Katz. FECs were multiplied by 12 or 24 to obtain EPG for duplicate or single Kato-Katz, respectively.

#### Mini-FLOTAC

The sample recipient of the Fill-FLOTAC was filled with fresh stool (2 gram) and thoroughly homogenized with 38 ml of flotation solution (saturated salt solution, specific density = 1.20), resulting in a final suspension volume of 40 ml. One ml of this suspension was then transferred into each of the two chambers of the Mini-FLOTAC device. The device was placed on a horizontal surface for 10 minutes after which the reading disk was turned clockwise using the key until it stopped. Both chambers of the Mini-FLOTAC device were screened for the presence of STH eggs. The number of eggs was multiplied by 10 to calculate the EPG (i.e., 2x 1 chamber of 1 ml was filled with a suspension containing 2 gram stool in a total volume of 40 ml).

#### FECPAK^G2^

The FECPAK^G2^ was performed as described previously [15]. Briefly, three gram of stool was homogenized in tap water (total suspension of 38 ml) in a Fill-FLOTAC device, after which it was transferred into a FECPAK^G2^ sedimenter to allow STH eggs to sediment. The following day, the supernatant was poured off and a saturated saline solution (specific density = 1.20) was added to the remaining slurry up to a total volume of 95 ml. The whole content of the sediment was then poured into a FECPAK^G2^ filtration unit from which two separate aliquots of 455 μl each were taken and transferred to two wells of a FECPAK^G2^ cassette. Following an accumulation step of 20 minutes, the cassette was placed in the Micro-I device for image capture. The device automatically imaged both wells and stored the images prior to uploading them to the FECPAK^G2^ server. Finally, a technician identified and counted the STH eggs present in the images using specialized software. These results were saved automatically for reporting and analysis. For FECPAK^G2^, a multiplication factor of 34 was used to calculate the EPG (i.e., 2x 1 well 0.455 ml was filled with a suspension containing 3 gram stool in a total volume of 95 ml).

#### Quality control of egg counting

A standard operating procedure for quality control (QC) of the egg counting has been described elsewhere [19, 20]. Briefly, egg counts of a subset (10%) of predefined samples were re-evaluated by a second independent reader for all methods. Because FECPAK^G2^ was newly introduced at all sites, the first hundred images were also re-analyzed. The egg counts (these do not correspond with the FECs, but they are the number of eggs counted under the microscope or in the FECPAK^G2^ images) obtained during QC were considered different due to false positive or negatives, when a QC egg count differed by >10 eggs from the original count (when original egg counts <100 eggs), or when QC count differed by more than 20% from the original egg count (when the original egg count ≥ 100). Discrepancies were resolved by a third reader to determine the final egg count [21]. A detailed analysis of the QC results will be described elsewhere.

### DNA extraction and qPCR protocol

After performing the different diagnostic microscopic methods, two aliquots of each stool sample were weighed and preserved in ethanol (96%) as described elsewhere [19, 20]. To minimize the risk of losing samples during shipment, the duplicate aliquots were shipped in two consecutive batches to the Laboratory of Parasitology (Ghent University, Ghent, Belgium). On arrival at Ghent University, samples were stored at 4°C and a 250 μl of one aliquot of ethanol preserved stool was used for DNA extraction. The detailed DNA extraction protocol (which includes disruption of STH eggs by means of bead beating and freeze-thawing) is validated (Ayana et al., under review) and described elsewhere [19, 20].

Reaction mixtures, primers, probes and cycling conditions of the qPCRs targeting *A*. *lumbricoides*, *T*. *trichiura*, *N*. *americanus* and *A*. *duodenale* are described elsewhere [20]. For each STH, a standard curve for absolute quantification was obtained by including a tenfold standard dilution series of genomic DNA extracted from worm/larvae material [20]. In each run, a positive control (a mixture of genomic DNA from each STH species) and a negative template control (Tris EDTA buffer instead of DNA template) and the negative DNA extraction control were included. In each sample, a known quantity of viral phocine herpes virus DNA was added to check for qPCR inhibition. All qPCR results were expressed in genome equivalents per ml of stool DNA extract (GE/ml). The limit of detection (LOD) and limit of quantification (LOQ) of each qPCR assay were determined as described elsewhere [20]. The LOD, LOQ and qPCR efficiencies are documented in S2 and S3 Infos. We did not assess LOD and LOQ for the *A*. *duodenale* qPCR because at the time of writing we did not have reference material for this species. Individual qPCR results for *N*. *americanus* and *A*. *duodenale* were taken together to obtain a final individual hookworm qPCR result. All analyses were performed on the RotorGene platform operated by the RotorGene Software Version 1.5 at the Laboratory for Medical Microbiology and Immunology (Elisabeth-Tweesteden Hospital, Tilburg, The Netherlands).

### Data analysis

#### Clinical sensitivity

The clinical sensitivity (or diagnostic sensitivity) is the proportion of individuals that are correctly identified as infected as determined by the diagnostic method in question. In absence of a gold standard (method that has a sensitivity and specificity of 100%), we considered the composite reference standard (CRS) [22] as a proxy for a gold standard to calculate the sensitivity of the different diagnostic methods. This CRS method classifies a subject as infected for an STH species if eggs or DNA is found at least one of the diagnostic methods and as negative if no eggs or DNA is found all methods. The sensitivity was determined assuming a 100% specificity of each method, as indicated by the morphology of the eggs or by the species-specific qPCR assays. The 95% confidence interval [95% CI] was determined based on bootstrap analysis (5,000 iteration). The significance of differences in sensitivity between a single Kato-Katz and the other methods was assessed by a permutation test (5,000 iterations) accounting for correlated test results and pair-wise comparison (Tukey method). To verify if a method fulfilled the criteria for sensitivity as put forward in the TPPs for use-case #1 and #2 (See Table 1), we assessed the sensitivity of the different methods across the different classes of infection intensity. Therefore, the infection intensity was based on the highest FEC across the microscopic methods (Kato-Katz, Mini-methods and FECPAK^G2^) applying the WHO thresholds listed in Table 2 [23]. To gain further insights in the potential use of the different methods in use-case#3, which regards settings of low-to-very low transmission, we further stratified the low infection intensity category into arbitrarily chosen intervals and assessed the sensitivity across these different levels of egg excretion.

**Table 2:** Fecal egg count thresholds defining low, moderate and heavy infection intensity [23]. The fecal egg counts are expressed as eggs per gram of stool.

	Low	Moderate	Heavy
***Ascaris***	1–4,999	5,000–49,999	≥50,000
***Trichuris***	1–999	1,000–9,999	≥10,000
**Hookworm**	1–1,999	2,000–3,999	≥4000

#### Intensity of infection

The agreement in FECs between a single Kato-Katz and the other microscopic methods was evaluated by permutation tests (5,000 iterations) based on both the Spearman correlation coefficients and the differences in mean FECs. Tukey’s method was applied for pair-wise comparisons within each STH species. The 95% CI was determined based on bootstrap analysis (5,000 iteration). In addition, the agreement between a single Kato-Katz and the other egg counting methods in categorizing the infection intensity into low, moderate and high was evaluated by Fleiss’ kappa statistic (κ_Fleiss_). The class of infection intensity was on the WHO threshold criteria described in Table 2. The value of this statistic indicates a slight (κ_Fleiss_ <0.2), fair (0.2 ≤ κ_Fleiss_ <0.4), moderate (0.4 ≤ κ_Fleiss_ <0.6), substantial (0.6 ≤ κ_Fleiss_ <0.8) and an almost perfect agreement (κ_Fleiss_ ≥ 0.8).

For qPCR, the agreement in DNA-concentration (expressed in GE/ml) and the FECs obtained by a single Kato-Katz was evaluated using a permutation test (10,000 iterations) based on the Spearman correlation coefficient. All statistical analyses were performed in R (Team, 2016).

## Results

### Demographics of the complete cases

**Fig 1:**
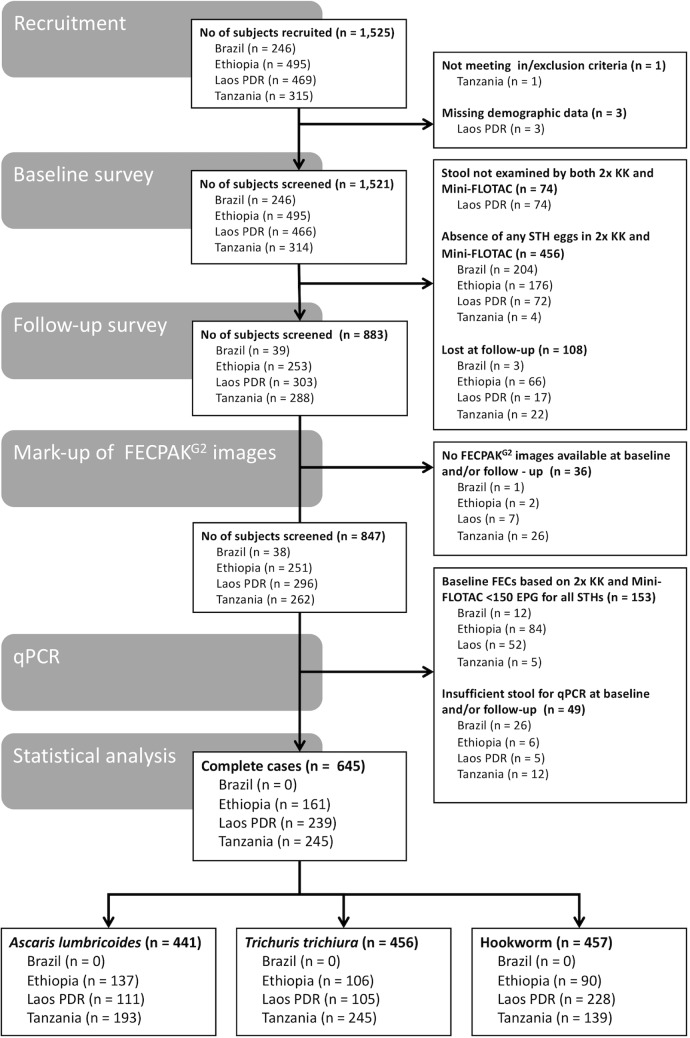
Number of subjects withheld at recruitment, baseline and follow-up, and for the statistical data analysis. STH: soil-transmitted helminth; n: number of subjects; FECs: fecal egg counts expressed in eggs per gram of stool (EPG), 2x KK: duplicate Kato-Katz.

Fig 1 represents the number of children withheld at recruitment, baseline and follow-up, and ultimately the number of complete cases for the statistical data analysis. Complete data was available for 645 children across the three study sites (Ethiopia: 161 children; Lao PDR: 239 children; Tanzania: 245 children). The overall sex ratio was 1:1.1 (310 males *vs*. 335 females). The median age (25^th^ (Q25) and 75^th^ quintile (75Q)) of the children equaled 11.0 years (9.0; 12.0). S4 Info describes the number of complete cases per school, sex ratio and age across the three study sites. Due to the nature of the school selection procedure (prioritization of schools where STH prevalence was expected to be moderate to high and premature discontinuation of recruitment when the prevalence of STH revealed to be low), the complete cases are not equally distributed across the schools. Rather the minority of the schools represent the majority of the complete cases. With the exception of Tanzania where females were proportionally more represented (sex ratio = 1:1.3), the sex ratio was approximately 1:1.0 in all study sites (Ethiopia: 1:1.1, Lao PDR: 1:0.9). In Ethiopia, the complete cases were slightly younger (9.0 years [8.0; 10.0]) than those in Lao PDR (12.0 years [11.0; 13.0]) and Tanzania (11.0 years [10.0; 12.0]).

### Clinical sensitivity

The clinical sensitivity of Kato-Katz (single and duplicate), Mini-FLOTAC, FECPAK^G2^ and qPCR for the diagnosis of *A*. *lumbricoides* (540 cases), *T*. *trichiura* (889 cases) and hookworm infections (675 cases) is shown in Table 3. For a single Kato-Katz, the clinical sensitivity equaled 71.9% for *A*. *lumbricoides*, 72.6% for hookworm and 88.1% for *T*. *trichiura*. Both a duplicate Kato-Katz and qPCR showed an equal or higher clinical sensitivity compared to a single Kato-Katz for all three STHs. For a double Kato-Katz, the increase in sensitivity was not significant (*A*. *lumbricoides*: 71.9% *vs*. 73.0%, *p* = 0.986; hookworms: 72.6% *vs*. 75.3%, *p* = 0.516; *T*. *trichiura*: 88.1% *vs*. 90.9%, *p* = 0.068). The sensitivity of qPCR compared to a single Kato-Katz was significantly higher for all STHs (*A*. *lumbricoides*: 71.9% *vs*. 90.0%, hookworms: 72.6% *vs*. 91.9%; *T*. *trichiura*: 88.1% *vs*. 94.7%, *p* <0.001). The Mini-FLOTAC method was significantly more sensitive for diagnosing *T*. *trichiura* infections (88.1% *vs*. 91.5%, *p* = 0.017), but detected significantly less *A*. *lumbricoides* infections (71.9% *vs*. 63.3%, *p* <0.001). For hookworm, no significant difference between a single Kato-Katz and Mini-FLOTAC was observed (72.6% *vs*. 73.9%, *p* = 0.920). FECPAK^G2^ was less sensitive compared to a single Kato-Katz for all STHs (*A*. *lumbricoides*: 71.9% *vs*. 58.9%, p = 0.003; hookworms: 72.6% *vs*. 52.4%, *p* <0.001; *T*. *trichiura*: 88.1% *vs*. 59.8%, *p* <0.001).

**Table 3:** The overall clinical sensitivity of five diagnostic methods for soil-transmitted helminth infections in stool. The Table summarizes the clinical sensitivity and corresponding 95% confidence intervals [95% CI] of Kato-Katz (single and duplicate), Mini-FLOTAC, FECPAK^G2^ and qPCR across all cases of *Ascaris* (n = 540), *Trichuris* (n = 889) and hookworms (n = 675). In addition, it reports the level of significance (*p*-value) for the hypothesis that the clinical sensitivity is different from that of a single Kato-Katz.

	*Ascaris* (n = 540)		*Trichuris* (n = 889)		Hookworm (n = 675)	
	Sensitivity (%) [95% CI]	*p*-value	Sensitivity (%) [95% CI]	*p*-value	Sensitivity (%) [95% CI]	*p*-value
**Single Kato-Katz**	71.9 [68.0; 75.6]	*_*	88.1 [86.0; 90.2]	*_*	72.6 [69.2; 76.0]	*_*
**qPCR**	90.0 [87.4; 92.4]	<0.001	94.7 [93.1; 96.1]	<0.001	91.9 [89.8; 93.8]	<0.001
**Duplicate Kato-Katz**	73.0 [69.1; 76.7]	0.986	90.9 [89.0; 92.7]	0.068	75.3 [71.9; 78.5]	0.516
**Mini-FLOTAC**	63.3 [59.3; 67.4]	<0.001	91.5 [89.5; 93.3]	0.017	73.9 [70.4; 77.2]	0.920
**FECPAK^G2^**	58.9 [54.6; 62.8]	0.003	59.8 [56.6; 63.1]	<0.001	52.4 [48.4; 56.1]	<0.001

**Fig 2:**
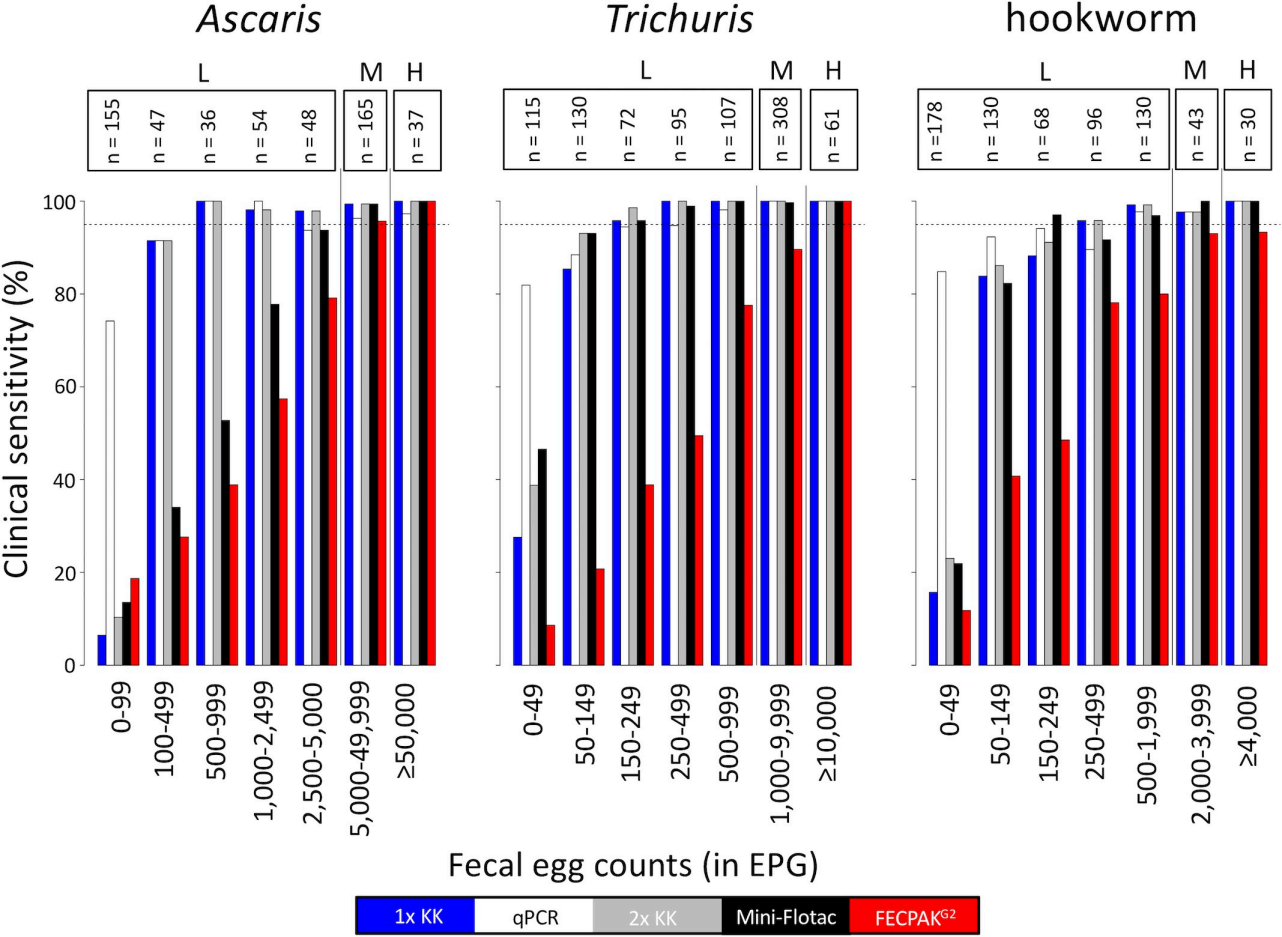
Clinical sensitivity of five diagnostic methods as a function of egg excretion. The bar plots represent the clinical sensitivity of single Kato-Katz (blue), qPCR (white), duplicate Kato-Katz (grey), Mini-FLOTAC (black) and FECPAK^G2^ (red) across seven infection intensity categories. The range in fecal egg counts (FECs; expressed as eggs per gram of stool (EPG)) is shown on the X-axis. These FECs correspond with the highest FECs across the microscopic methods (Kato-Katz, Mini-FLOTAC and FECPAK^G2^). The Y-axis represent the clinical sensitivity in percentage. The number samples for each infection category (n) is shown above the bars. The WHO defined classes of infections intensities (low (L), moderate (M) and high (H)) are shown on top. The dotted horizontal line represents a clinical sensitivity of 95%.

Fig 2 illustrates the change in clinical sensitivity as a function of egg excretion (= highest FEC across the microscopic methods). The clinical sensitivity of each of the diagnostic methods increased as a function of increasing egg excretion. However, this trend of increased clinical sensitivity over increasing FECs differed considerably across both diagnostic methods and STH species. In contrast to qPCR that shows a clinical sensitivity of at least 70%, the microscopic methods were not able to detect more than 50% of the lowest levels of egg excretion (*A*. *lumbricoides*: FEC <100 EPG; *T*. *trichiura*: FEC <50 EPG; hookworm: FEC <150 EPG). Both a single and duplicate Kato-Katz already showed a clinical sensitivity comparable to qPCR when levels of egg excretion were at least 50 EPG for *T*. *trichiura*, 100 EPG for *A*. *lumbricoides* and 150 EPG for hookworm. For Mini-FLOTAC, the trend of increasing clinical sensitivity over increasing egg excretion was comparable to that of Kato-Katz, except for *A*. *lumbricoides*. For this STH species, Mini-FLOTAC only showed a clinical sensitivity comparable to qPCR when the egg excretion ≥2,500 EPG. FECPAK^G2^ only revealed a clinical sensitivity comparable to qPCR for the moderate and heavy infection intensities. With the exception of FECPAK^G2^, all diagnostic methods detected at least 95% of the moderate and heavy intensity infections. For FECPAK^G2^, this level of clinical sensitivity was not achieved for both hookworm (moderate infections: 93.0% and heavy intensity: 93.3%) and *T*. *trichiura* infections (moderate infections: 89.6%).

The clinical sensitivity of a single and duplicate Kato-Katz, Mini-FLOTAC, FECPAK^G2^ and qPCR for the detection of low intensity infections is shown in Table 4. Both a duplicate Kato-Katz (*A*. *lumbricoides*: 57.4%, *p* = 0.978; *T*. *trichiura*: 84.4%, *p* = 0.060; hookworm: 72.4%, *p* = 0.512) and qPCR (*A*. *lumbricoides*: 86.2%, *p* <0.001; *T*. *trichiura*: 91.0%, *p* <0.001; hookworm: 91.0%, *p* <0.001) were equally or more sensitive compared to a single Kato-Katz for all three STH species (*A*. *lumbricoides*: 55.6%; *T*. *trichiura*: 79.6%; hookworm: 69.4%; Table 4). Mini-FLOTAC was equally or more sensitive compared to a single Kato-Katz for *T*. *trichiura* (85.6%, *p* = 0.013) and hookworm (70.8%, *p* = 0.952), but not for *A*. *lumbricoides* (42.1%, p <0.001). FECPAK^G2^ was less sensitive compared to a single Kato-Katz for all three STH species (*A*. *lumbricoides*: 36.8%; *T*. *trichiura*: 37.5% hookworm: 47.5%, *p* <0.001).

**Table 4:** The clinical sensitivity of five diagnostic methods for low infection intensity STH infections. The Table summarizes the clinical sensitivity and corresponding 95% confidence intervals [95% CI] of Kato-Katz (single and duplicate), Mini-FLOTAC, FECPAK^G2^ and qPCR across low intensity infections of *Ascaris* (n = 340), *Trichuris* (n = 519) and hookworms (n = 602). In addition, it reports the level of significance (*p*-value) for the hypothesis that the clinical sensitivity is different from that of a single Kato-Katz.

	*Ascaris* (n = 340)		*Trichuris* (n = 519)		Hookworm (n = 602)	
	Sensitivity (%) [95% CI]	*p*-value	Sensitivity (%) [95% CI]	*p*-value	Sensitivity (%) [95% CI]	*p*-value
**Single Kato-Katz**	55.6 [50.6; 60.9]		79.6 [76.0; 82.9]		69.4 [65.8; 73.1]	
**qPCR**	86.2 [82.4; 89.7]	<0.001	91.0 [88.5; 93.5]	<0.001	91.0 [88.7; 93.2]	<0.001
**Duplicate Kato-Katz**	57.4 [52.4.1; 62.6]	0.978	84.4 [81.2; 87.5]	0.060	72.4 [68.9; 75.9]	0.512
**Mini-FLOTAC**	42.1 [36.8; 47.4]	<0.001	85.6 [82.5; 88.5]	0.013	70.8 [66.9; 74.3]	0.952
**FECPAK^G2^**	36.8 [31.8; 42.1]	<0.001	37.5 [33.3; 41.7]	<0.001	47.5 [43.5; 51.5]	<0.001

### Infection intensity measured by microscopic methods

**Fig 3:**
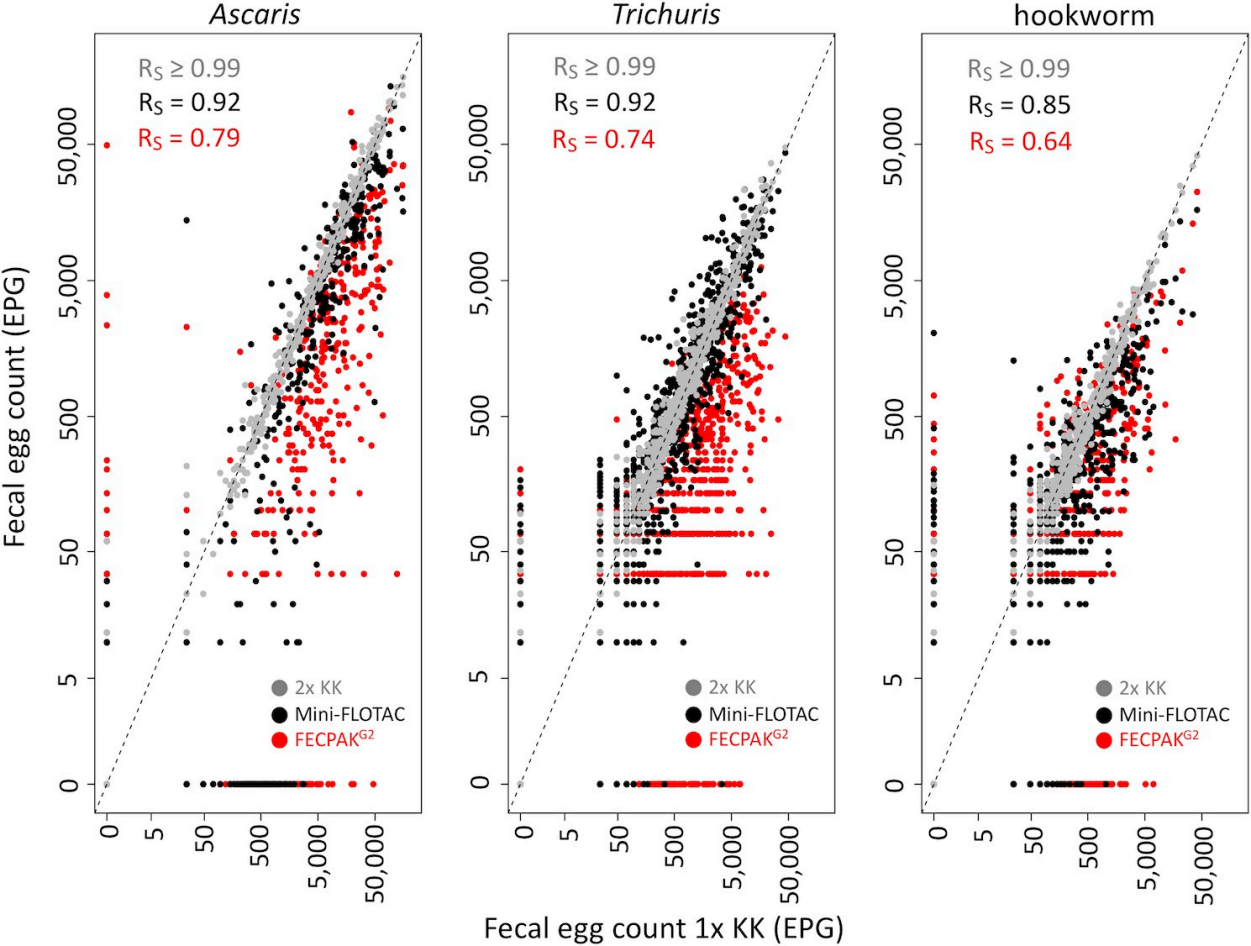
Agreement in fecal egg counts by the four microscopic methods. The scatterplots illustrate the agreement in fecal egg counts (FECs, expressed in eggs per gram of stool (EPG) based on a single Kato-Katz (1x KK) with those obtained with a duplicate Kato-Katz (grey dots), Mini-FLOTAC (black dots) and FECPAK^G2^ (red dots) for *A*. *lumbricoides* (n = 540), *T*. *trichiura* (n = 889) and hookworm (n = 675). In each panel, the Spearman’s correlation coefficient (R_s_) is given. The striped diagonal line represents the line of equivalence.

Fig 3 illustrates the agreement in FECs obtained between a single Kato-Katz and the other microscopic methods for each of the STHs. Overall, a significant positive correlation was observed for each pair-wise comparison for each STH species. The highest correlation coefficients were observed between a single and a duplicate Kato-Katz (R_s_ >0.99), the lowest between a single Kato-Katz and FECPAK^G2^ (R_s_: 0.64–0.79). For Mini-FLOTAC, the coefficients ranged from 0.85 to 0.92.

Generally, both Mini-FLOTAC and FECPAK^G2^ resulted in lower FECs compared to a single Kato-Katz (the majority of the dots in Fig 3 are located under the line of equivalence), and these differences in FECs are represented in Table 5. The mean FECs based on a single Kato-Katz equaled 10,000 EPG for *A*. *lumbricoides*, 1,917 EPG for *T*. *trichiura* and 833 EPG for hookworms. Compared to a single Kato-Katz, a duplicate Kato-Katz showed comparable FECs (*A*. *lumbricoides*: 10,000 EPG *vs*. 10,288 EPG, *p* = 0.970; *T*. *trichiura*: 1,917 *vs*. 2,016 EPG, *p* = 0.901; hookworms: 833 *vs*. 819 EPG, *p* = 0.993). Mini-FLOTAC resulted in comparable FECs for *T*. *trichiura* (1,918 *vs*. EPG 1,838, *p* = 0.947), but provided significant lower FECs for both *A*. *lumbricoides* (10,000 *vs*. 6,404 EPG, *p* <0.001) and hookworms (833 *vs*. 366 EPG, *p* <0.001). FECPAK^G2^ resulted in significant lower FECs for all STH species (*A*. *lumbricoides*: 10,000 *vs*. 3,266 EPG; *T*. *trichiura*: 1,918 *vs*. 286 EPG; hookworms: 833 *vs*. 275 EPG, *p* <0.001).

**Table 5:** The mean fecal soil-transmitted helminth egg counts based on four microscopic methods. The arithmetic mean of fecal egg counts (FECs; expressed as eggs per gram of stool) were calculated for each of the four microscopic methods (Kato-Katz (single and duplicate), Mini-FLOTAC and FECPAK^G2^).

	*Ascaris* (n = 540)		*Trichuris* (n = 889)		Hookworm (n = 675)	
	Mean FECs (EPG) [95% CI]	*p*-value	Mean FECs (EPG) [95% CI]	*p*-value	Mean FECs (EPG) [95% CI]	*p*-value
**Single Kato-Katz**	10,000 [8,335; 11,808]		1,917 [1,664; 2,178]		833 [637; 1,071]	
**Duplicate Kato-Katz**	10,288 [8607; 12,100]	0.970	2,016 [1,738; 2,305]	0.901	819 [622; 1,058]	0.993
**Mini-FLOTAC**	6,404 [5,332; 7,554]	<0.001	1,838 [1,592; 2,094]	0.947	366 [293; 452]	<0.001
**FECPAK**^**G2**^	3,266 [2,513; 4,124]	<0.001	286 [246; 328]	<0.001	275 [202; 372]	<0.001

**Fig 4:**
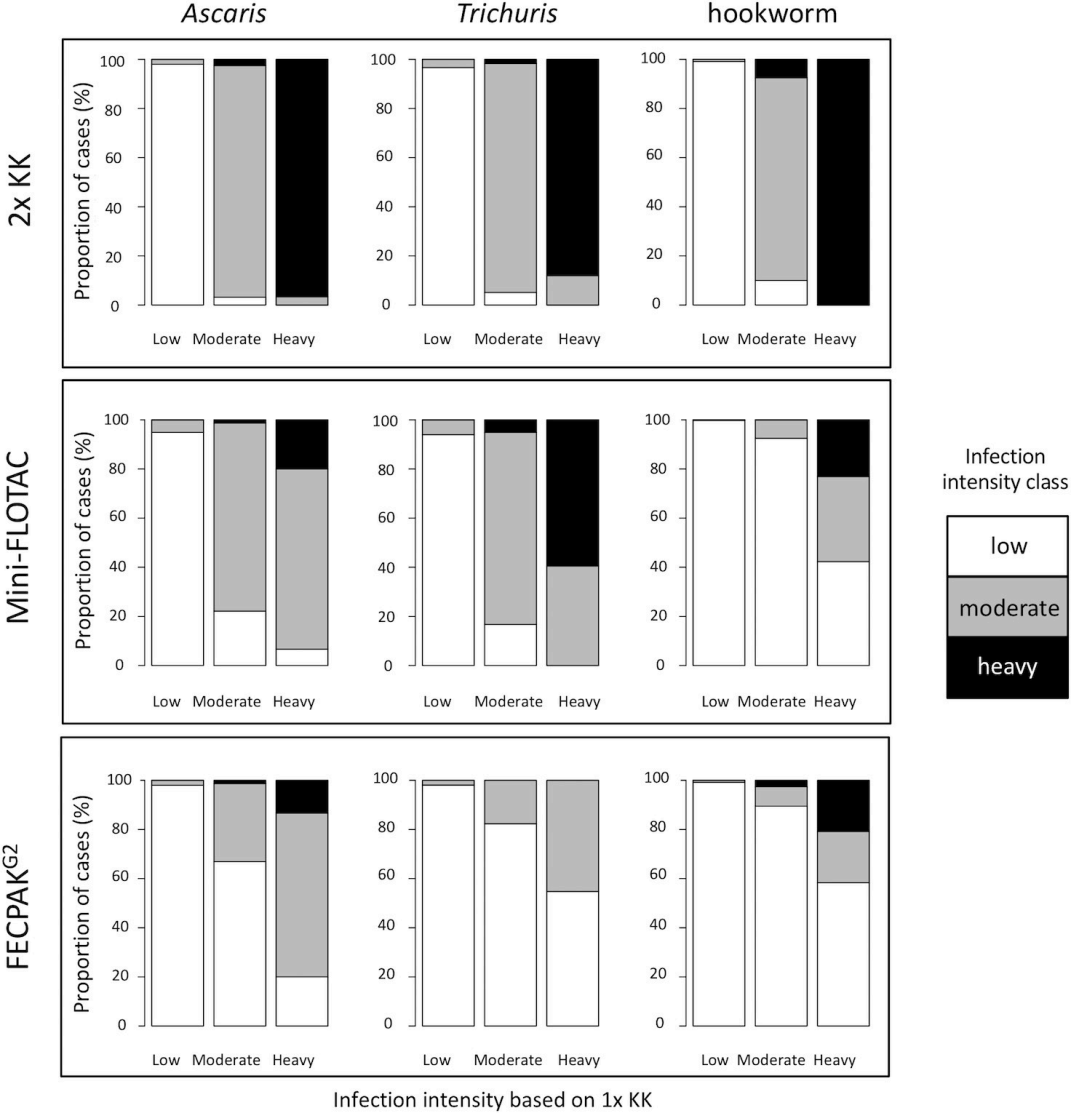
Agreement in infection intensity classification by four microscopic method. Each bar represents all cases classified by a single Kato-Katz (1x KK; reference method) into low, moderate or heavy infection intensity using the WHO threshold criteria (Table 2). The Y-axis of each bar represents the proportion of these cases that are categorized as low, moderate and heavy intensity infections by means of a duplicate Kato-Katz (2x KK; top row plots), Mini-FLOTAC (middle row plots) and FECPAK^G2^ (bottom row plots). The white color indicates cases classified as low infection intensity, grey indicates cases classified as moderate infection intensity and black indicates cases categorized as heavy infection intensity, by either duplicate Kato-Katz, Mini-FLOTAC or FECPAK^G2^. There is a full agreement in classifying the intensity of infection with the reference method when the bars representing low, moderate and high infection intensities by the reference method are completely white, grey and black, respectively. As an example, of all heavy intensity hookworm infections, only approximately 20% are classified correctly as heavy when using the FECPAK^G2^, and approximately 60% and 20% are misclassified as low and moderate, respectively.

These differences in FECs across microscopic methods also resulted in discrepancies in classifying the intensity of infection as low, moderate and heavy (Fig 4). The agreement in classifying the intensity of infection was almost perfect between a single and duplicate Kato-Katz (κ_*A*. *lumbricoides*_ = 0.96; κ_hookworm_ = 0.96; κ_*T*. *trichiura*_ = 0.93, *p* <0.001). For Mini-FLOTAC, the agreement was moderate for hookworms (κ = 0.46, *p* <0.001), substantial for *Ascaris* (κ = 0.69, *p* <0.001) and almost perfect for *Trichuris* (κ = 0.80, *p* <0.001). For FECPAK^G2^, the agreement was slight for *T*. *trichiura* (κ = 0.18, *p* <0.001) and fair for both hookworms (κ = 0.37, *p* <0.001) and *A*. *lumbricoides* (κ = 0.39, *p* <0.001). As illustrated by Fig 4, both Mini-FLOTAC and FECPAK^G2^ often assigned the samples to a lower level of infection intensity.

### Infection intensity measured by qPCR

**Fig 5:**
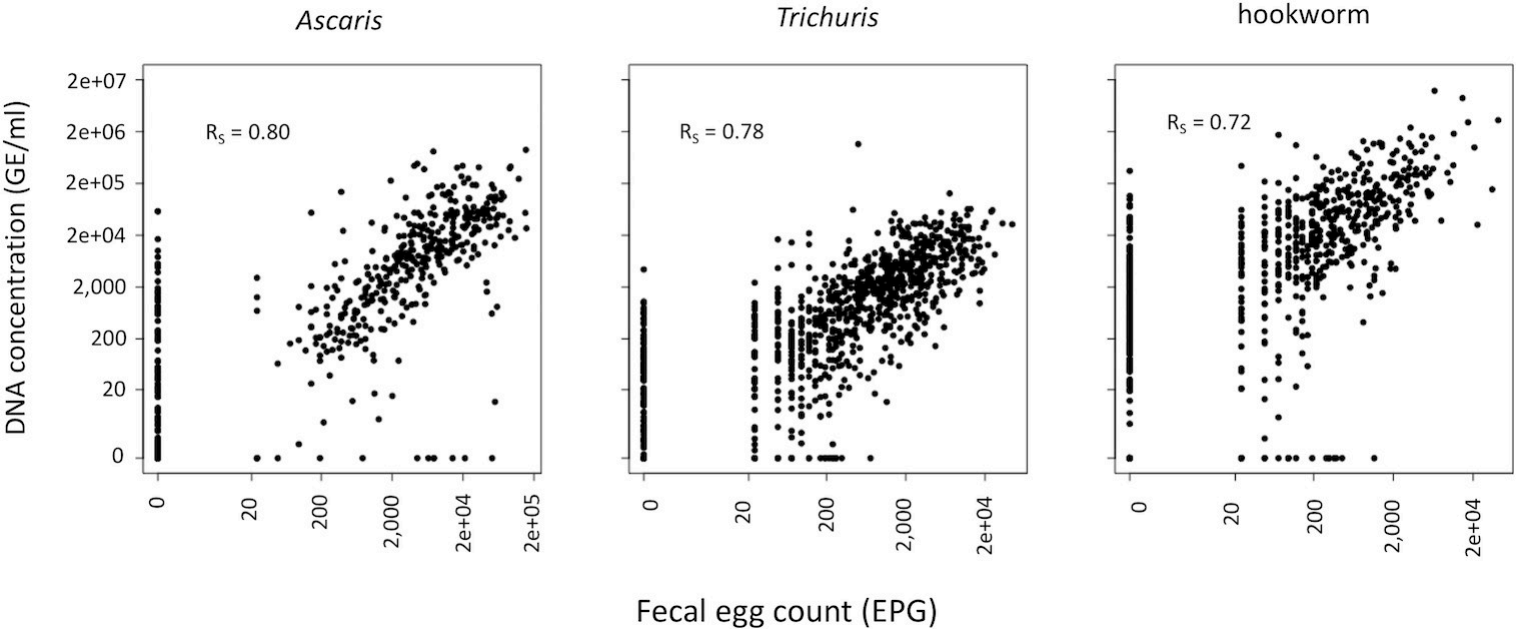
Agreement in fecal egg counts by Kato-Katz and genomic concentration by qPCR. The scatterplots illustrate the agreement in fecal egg counts (FECs; expressed in eggs per gram of stool (EPG)) based on a single Kato-Katz and the DNA concentration (expressed in number of genome equivalents per ml (GE/ml)) based on qPCR for *Ascaris* (n = 540), *Trichuris* (n = 889) and hookworm (n = 675). In each panel, the Spearman’s correlation coefficient (R_S_) is shown.

When the infection intensity was measured by means of qPCR (expressed in GE/ml), the mean intensity equaled 132,201 GE/ml (95% CI: 91,674; 185,809) for hookworms, 27,528 GC/ml (95% CI: 21,112; 34,773) for *A*. *lumbricoides* and 5,547 GE/ml (95% CI: 3,882; 8,538) for *T*. *trichiura*. Fig 5 illustrates the agreement in FECs obtained between a single Kato-Katz and the corresponding intensity expressed in GE/ml for the three STH species. A positive significant correlation was observed for all STHs (R_s_: 0.72–0.80, *p* <0.001).

## Discussion

In the present study, we evaluated the diagnostic performance of a single and duplicate Kato-Katz, Mini-FLOTAC, FECPAK^G2^ and qPCR and explored which of these different diagnostic methods met the recently developed TPPs. To our knowledge, this is the most extensive study so far reporting a head-to-head comparison of five different STH diagnostic methods, performed in the context of three ALB drug efficacy trials in three different STH endemic countries with varying levels of STH infections.

### qPCR outperforms microscopic methods in clinical sensitivity

Of all diagnostic methods, qPCR had the highest clinical sensitivity for all three STHs. This finding is largely in line with previous studies that compared the qPCR with microscopic methods (direct smear, wet preparation, Kato-Katz (single and duplicate on single or multiple stool samples over consecutive days), McMaster and/or sodium nitrate flotation [24–36]). However, in contrast to our findings and those listed above, Knopp and coworkers (2014) found a lesser clinical sensitivity of qPCR (73.6%) compared to microscopic methods (83.3% by means of FLOTAC and 75.0% by means of Kato-Katz) for the detection of hookworm in Tanzania, although not regarded as significant [37]. This discrepancy might be due to the fact that only a qPCR assay specific for *N*. *americanus* was performed, and not for *A*. *duodenale*. Studies have shown that in East-Africa, *A*. *duodenale* is prevalent as the minority hookworm species (as found in the present study and by Albonico and coworkers (2012) [38]) or as the most prevalent hookworm species [39, 40].

### Flotation based methods fail to detect *A*. *lumbricoides*

Mini-FLOTAC and FECPAK^G2^, both flotation-based methods, were significantly less sensitive for the diagnosis of *A*. *lumbricoides* compared to a single Kato-Katz. Although FECPAK^G2^ shows a poor clinical sensitivity across all STHs, which suggests a more technical cause, this was not the case for Mini-FLOTAC. This method had a slightly higher clinical sensitivity compared to a single Kato-Katz for *T*. *trichiura* and hookworm but had a significantly lower clinical sensitivity for *A*. *lumbricoides*. The lower clinical sensitivity of Mini-FLOTAC for the detection of *A*. *lumbricoides* might be explained by the use of saturated salt solution as flotation solution. Indeed, it has been shown by Barda and coworkers (2014) that when using a flotation solution with a higher specific density than saturated salt solution (i.e. zinc sulphate, specific density = 1.35), Mini-FLOTAC was more sensitive (87.1%) compared to using saturated salt solution (61.3%) or Kato-Katz (84.4%) for the detection of *A*. *lumbricoides* [41]. Barda and colleagues did not observe the trend for the other STHs, although the number of cases for these were low [41]. The lesser clinical sensitivity for *A*. *lumbricoides* found in our study and the findings of Barda and coworkers might also be further explained by the presence of unfertilized *A*. *lumbricoides* eggs. These unfertilized eggs have a higher density compared to fertilized *A*. *lumbricoides* eggs [42], hence may require a flotation solution with higher density for optimal detection. Although this hypothesis is not new [43], it has not yet been supported by data. In this study, we did not differentiate the fertilized from unfertilized eggs with Kato-Katz, and hence we too were not able to provide the necessary evidence to support the hypothesis. There is no flotation solution that is optimal for all STHs. For example, saturated salt solution is more optimal for hookworms, whereas zinc sulphate (specific density = 1.35) is more optimal for *Ascaris* [44]. However, it is clear that recommending two different flotation solutions for an optimal detection across all STHs will have both financial and logistic consequences when applied on a large number of samples [45].

### FECPAK^G2^ is the least sensitive method

The FECPAK^G2^ method showed the lowest clinical sensitivity for all STHs, confirming previous findings [46]. These results are not that unexpected as Ayana and coworkers (2018), who optimized the FECPAK^G2^ methodology for the detection of human STHs, already found a suboptimal recovery rate of STHs eggs using this platform [15]. Furthermore, this FECPAK^G2^ method used a first version of a product currently undergoing extensive research and development aiming to further optimize the diagnostic performance. Because automated egg counting and species differentiation is one of the optimal criteria for TPPs #1 and 2 (Table), we also explored whether the FECPAK^G2^ images could be analyzed with a downstream egg counting software (Helminth Egg Automated Detector, (HEAD); [47]). However, the resolution of the images was insufficient to be compatible with the HEAD software (See S5 Info). Improvements with regards to the sample preparation and sample imaging are currently underway aiming to increase egg recovery, close the gaps in both clinical sensitivity and egg counting with the other diagnostic methods and to eventually automate the egg counting.

### All diagnostic methods show high sensitivity for moderate to heavy intensity infections

It is important to note that the sensitivity changed over the level of egg excretion, all methods showing a sensitivity of >90% when the intensity of infection was moderate to heavy. Although it is commonly accepted that a single Kato-Katz fails to detect low infection intensity [10], this level of infection intensity is represented by a wide range of FECs (*A*. *lumbricoides*; 1–4,999 EPG; *T*. *trichiura*: 1–999 EPG; hookworm: 1–1,999 EPG). The present study showed that a single and a duplicate Kato-Katz are only missing the very low levels of infection intensity (*A*. *lumbricoides*; 1–99 EPG; *T*. *trichiura*: 1–49 EPG; hookworm: 1–49 EPG), suggesting that the method may have more potential than currently accepted. The same trends are true for Mini-FLOTAC, except for *A*. *lumbricoides*.

### Current WHO infection intensity thresholds cannot be applied across microscopic methods

Although the FECs obtained by the different microscopic methods for the different STHs correlated well with the single Kato-Katz, they were often significantly lower compared to the reference method. This implies that current WHO infection intensity thresholds, which are largely used in combination with the WHO recommended Kato-Katz, cannot be applied across the other microscopic methods. Indeed, Mini-FLOTAC or FECPAK^G2^ assigned a substantial proportion of the study population into lower levels of infection intensity. As the proportion of a population that harbors moderate and heavy intensity infections is a key indicator used in STH control programs for decision making, our findings highlight the urgent need for adjusted infection intensity thresholds for diagnostic methods alternative to Kato-Katz. The qPCR quantitative output (expressed in GE/ml) correlated well with the microscopic methods. However, given the different units between qPCR and the microscopic methods, we could not apply current thresholds for qPCR, highlighting the need to define these thresholds for qPCR too.

### Matching the diagnostic methods against the TPPs

Our study was performed in three different settings of moderate (prevalence of any STH >20%) or high (prevalence of any STH >50%) endemic areas. These settings are the envisioned settings for use-cases #1 and #2 and their corresponding TPPs. Our data suggest that all diagnostics evaluated in current study, except FECPAK^G2^, meet the TPPs #1 and #2 clinical sensitivity criteria for the moderate and heavy intensity infections (see Table 1). For the low intensity infections, only duplicate Kato-Katz and qPCR met the clinical sensitivity criteria for TPPs #1 and #2, FECPAK^G2^ and Mini-FLOTAC did not show a sensitivity equal or superior to a single Kato-Katz for all STHs. Strictly applying the criteria put forward in TPP #1 and TPP #2, qPCR is ruled out as these TPPs are targeting microscopic methods (optimistic, automated with autonomous counting of the different STHs).

Use-case #3 relates to programs that want to verify if transmission has been interrupted, and hence it applies to low-to-very low prevalence settings. Although the corresponding TPPs #3 favor non-stool-based technologies, the superior sensitivity of qPCR and simultaneous detection of multiple helminth infections makes it an option for use-case #3. This is particularly useful since at present there are no non-stool-based alternatives available and a theoretical qPCR decision prevalence threshold for breaking transmission has been developed [48] and which is currently being used in the DEWORM3 trials [49]. Furthermore, pooling of stool samples (an optimistic sample criteria for TPPs #3) has already been shown to be a promising sampling strategy for the detection and quantification of STH, *Schistosoma mansoni* using microscopic methods [50–53], and has also been piloted for qPCR in the present study (results will be published elsewhere).

Currently, there is a growing momentum to integrate different helminth control programs as a more cost-effective approach compared to stand-alone control programs [54]. Examples of other helminth infections with major public health impact are schistosomiasis [55], strongyloidiasis [56] and taeniasis [57]. This vision is also reflected in TPPs #3, stating that assays should ideally also detect *Schistosoma* and *Strongyloides* species, which is possible with qPCR. Indeed, although the current study focused on STH, our multiplex qPCR assays also detected and quantified *Schistosoma* spp, *Strongyloides stercoralis* and *Taenia* spp. (S6 Info). In a subset of the samples the prevalence of these parasites was 3.5% for *Schistosoma* spp, 3.5% for *S*. *stercoralis* and 2.7% for *Taenia* spp.

### Challenges for implementation of qPCR in PC programs

Considerable obstacles are currently hampering qPCR to be truly considered as a(n) (*ad interim*) tool for use-case #3, i.e. (i) throughput and cost, (ii) standardization and (iii) quality assurance. First, programs that aim to confirm a break in transmission will need large scale surveys as the prevalence threshold defining the transmission breakpoint is anticipated to lie within the low-to-very low prevalence settings [11]. Although TPP #3 requirements do not define the throughput needed, currently, the throughput of qPCR is unlikely to be cost-effective for use in such large surveys because of the relatively high workload from sampling to reporting. However, the current limited throughput of qPCR arises from both the sampling strategy and the pre-analytical phase (i.e., DNA extraction), and not from the qPCR assay itself. For example, some qPCR platforms can easily process over 1,500 DNA samples within the hour [58]. Pooling of stool samples, as previously mentioned, may be cost-saving strategy, but this should be further explored in field conditions. Regarding the pre-analytical phase, expensive and time-consuming DNA extraction and purification procedures are required to remove inhibitors that negatively influencing qPCR present in stool samples and to lyse the STH eggs (Ayana et al., under review). The emergence of PCR DNA polymerases that are resistant to these inhibitors are therefore promising as these could allow to reduce the time of the DNA extraction procedures by omitting purification steps [59, 60].

Secondly, whereas Kato-Katz protocols are globally largely comparable in terms of reagents, procedures and reporting of the results, qPCR protocols are yet to be standardized. Indeed, qPCR for the detection and quantification of STHs is an umbrella term hosting a variety of methodologies both in terms of platforms, chemistries, target species [14], but most importantly, analysis and reporting. For example, different units such as Cq, Ct, genomic DNA concentration and plasmid DNA concentration are used to report quantitative results [34, 39, 61, 62]. Furthermore, the quantitative Cq values (currently the most widely used unit in the STH field) can be obtained by different analytical procedures which are laboratory specific. This lack of standardized analysis and reporting of qPCR results therefore impedes inter-laboratory comparisons of quantitative results. Consensus guidelines that deal with this issue would benefit the STH field.

Third, although qPCR includes both internal controls and external negative and positive controls as part of the quality control process (a minimum requirement set for TPPs #3), currently, there is no quality assurance. A key component of quality assurance is external quality assurance (EQA, often referred to as proficiency testing), a system for objectively checking a laboratory’s performance using an external agency or facility. Currently, such EQA for the detection (and quantification) of STHs by means of qPCR is lacking, but has been developed and pilot tested [63]. The results of this study will be published elsewhere.

### Conclusions

Compared to the WHO recommended Kato-Katz, only qPCR had a higher sensitivity for all STHs. This higher sensitivity was only due to the higher sensitivity in the very low infection intensities, highlighting that the diagnostic performance of a single Kato-Katz is underestimated by the scientific community. Both Mini-FLOTAC and FECPAK^G2^ differed markedly in FECs and led to falsely classifying the infection into lower levels of intensity, highlighting the need for method specific infection intensity thresholds. So far, Kato-Katz is the only method that meets the criteria for application in the planning, monitoring and evaluation phase of an STH program, whereas qPCR is the only method that could be considered in the phase that aims to seek confirmation for cessation of program.

## Supporting information

S1 Info Inclusion and exclusion criteria endorsed during the recruitment of participants for the field trials. (PDF)

S2 Info Performance characteristics of the different qPCR assays. (PDF)

S3 Info Limit of detection and limit of quantification of the qPCR assays. (PDF)

S4 Info Number of complete cases per school, sex and age across the three study sites. (PDF)

S5 Info Report analysis of FECPAK^G2^ images by HEAD software. (PDF)

S6 Info Prevalence of helminths (other than *Ascaris*, *Trichuris* and hookworm) as determined by qPCR in the three study sites. (PDF)

S7 Info Complete data set. (XLSX)
