# Supplementary material for: Diagnostic performance of a single and duplicate Kato-Katz, Mini-FLOTAC, FECPAKG2 and qPCR for the detection and quantification of soil-transmitted helminths in three endemic countries
Source: PLoS Negl Trop Dis. 2019 Aug 1;13(8):e0007446. doi: 10.1371/journal.pntd.0007446 (PMC6675048; doi:10.1371/journal.pntd.0007446)
Supplement: S1 Info — (PDF) [file pntd.0007446.s001.pdf]

S1 Info: Inclusion and exclusion criteria endorsed during the recruitment of participants for the field trials

Inclusion criteria	Exclusion criteria
• Subject is 5-14 years of age<br>• Subject is otherwise in healthy condition (based on medical history and physical examination)<br>• Parent(s)/guardians of subject signed an informed consent document indicating that they understand the purpose of and procedures required for the study and that they are willing to have their child participate in the study<br>• Subject of ≥5 years has assented to participate in the study<br>• Subject of ≥12 years has signed an informed consent document indicating that they understand the purpose of the study and procedures required for the study, and are willing to participate in the study<br>• Subject has provided a stool sample of at least 9 grams.	• Subject has active diarrhoea (defined as the passage of 3 or more loose or liquid stools per day) at baseline or follow-up.<br>• Subject has an acute medical condition or is experiencing a severe concurrent medical condition<br>• Subject has a known hypersensitivity to albendazole or mebendazole<br>• Subject has received anthelmintic treatment within 90 days prior to the start of the treatment<br>• Subject vomited within 4 hours following drug ingestion.<br>• Subject is not able to provide a stool sample of min 9 grams at baseline or follow-up.<br>• Subject has not swallowed the entire tablet.
