# Supplementary material for: Diagnostic performance of a single and duplicate Kato-Katz, Mini-FLOTAC, FECPAKG2 and qPCR for the detection and quantification of soil-transmitted helminths in three endemic countries
Source: PLoS Negl Trop Dis. 2019 Aug 1;13(8):e0007446. doi: 10.1371/journal.pntd.0007446 (PMC6675048; doi:10.1371/journal.pntd.0007446)
Supplement: S2 Info — (PDF) [file pntd.0007446.s002.pdf]

S2 Info. Performance characteristics of the different qPCR assays. qPCR were performed in a total volume of 25 µl.

qPCR assay	Efficiency (%)	LOD		LOQ	
		GE/ml	GE/reaction	GE/ml	GE/reaction
A. lumbricoides	108.1	44.7	1.12	79.4	1.99
T. trichiura	97.4%	8.5	0.21	42.7	1.07
N. americanus	98.0%	14.5	0.36	213.8	5.35
