# Supplementary material for: Diagnostic performance of a single and duplicate Kato-Katz, Mini-FLOTAC, FECPAKG2 and qPCR for the detection and quantification of soil-transmitted helminths in three endemic countries
Source: PLoS Negl Trop Dis. 2019 Aug 1;13(8):e0007446. doi: 10.1371/journal.pntd.0007446 (PMC6675048; doi:10.1371/journal.pntd.0007446)

S3 Info. Limit of detection and limit of quantification of the qPCR assays. Limit of detections (LODs) are shown in the top row, limit of quantifications (LOQs) are shown in the bottom row. Horizontal dashed lines represent the 95% probability line (LOD) and the 35% coefficient of variation line (LOQ). Interpolation to the $\log_{10}$ concentration axis gives the LOD and LOQ, respectively. The LODs and LOQs have been assessed as described elsewhere (www.starworms.org/documents).

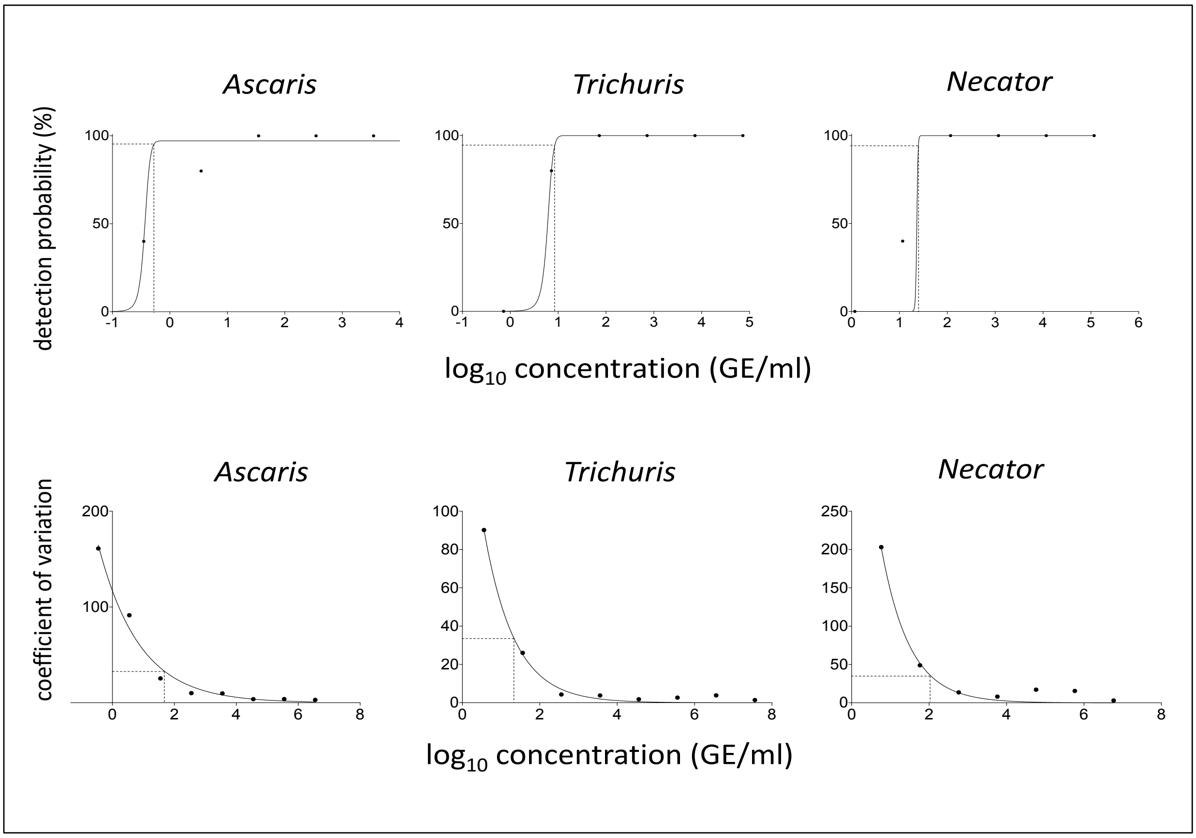

Supplement: S3 Info — (PDF) [file pntd.0007446.s003.pdf]
