# Supplementary material for: Diagnostic performance of a single and duplicate Kato-Katz, Mini-FLOTAC, FECPAKG2 and qPCR for the detection and quantification of soil-transmitted helminths in three endemic countries
Source: PLoS Negl Trop Dis. 2019 Aug 1;13(8):e0007446. doi: 10.1371/journal.pntd.0007446 (PMC6675048; doi:10.1371/journal.pntd.0007446)
Supplement: S4 Info — (PDF) [file pntd.0007446.s004.pdf]

S4 Info: Number of complete cases per school, sex and age across the three study sites

	Ethiopia (n = 161)	Laos (n = 239)	Tanzania (n = 245)
**School ID**			
01	143	13	64
02	18	33	21
03		151	85
04		35	75
05		7	
**Sex**			
Female	83	115	137
Male	78	124	108
**Age (in years)**			
6	6	10	0
7	20	11	20
8	35	12	23
9	31	13	14
10	33	8	28
11	13	23	82
12	8	47	52
13	14	62	19
14	1	53	7
