# Supplementary material for: Diagnostic performance of a single and duplicate Kato-Katz, Mini-FLOTAC, FECPAKG2 and qPCR for the detection and quantification of soil-transmitted helminths in three endemic countries
Source: PLoS Negl Trop Dis. 2019 Aug 1;13(8):e0007446. doi: 10.1371/journal.pntd.0007446 (PMC6675048; doi:10.1371/journal.pntd.0007446)
Supplement: S5 Info — (PDF) [file pntd.0007446.s005.pdf]

S5 Info. Report analysis of FECPAK images by HEAD software

Analysis of FECPAK^{G2} images by HEAD

Based on the images provided by the FECPAK^{G2} group, it was not possible to obtain the scale from the graduated slide after processing it both with Matlab and the autoscaling module of HEAD. However, the scaled image uploaded to OneDrive had a different scale than needed and had an uneven illumination (Background); thus, it was divided and later analyzed by sub-sections with the latest version of HEAD. The following is a summary of the main findings.

Scaled image processing

Image A shows the detection and classification of the whole scaled image. Based on the results, HEAD was not capable of identifying the probable helminth eggs as the software is not trained for these structures as they are beyond the metrics of the system. As a result, the scaled image provided was tested by dividing it into different sections and observed a similar egg size as that used by HEAD.

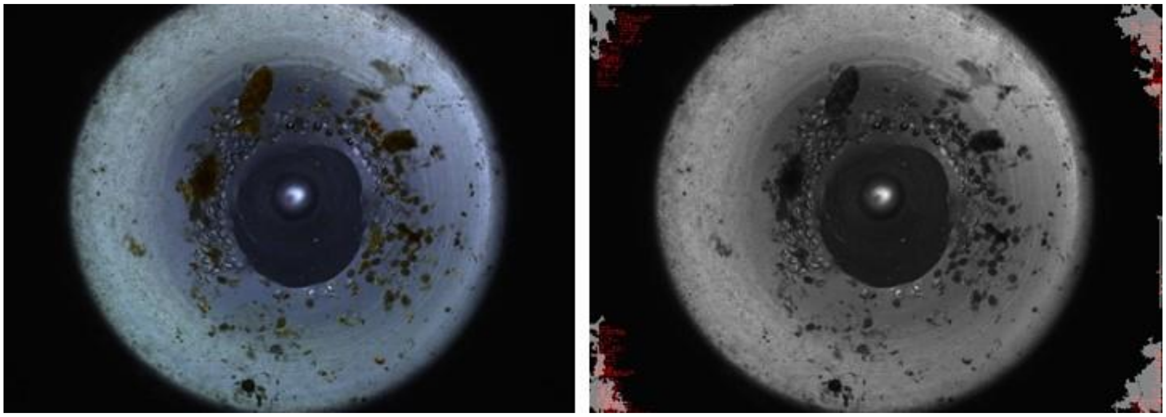

Image A. **FECPAK^{G2} image processed with HEAD.**

Object segmentation

Most of the well-focused eggs in the image sections could be segmented by HEAD. The segmentation process did not present major problems with probable eggs that were not focused, or with eggs that were surrounded by a considerable amount of debris. This can be seen in Images b, c and d where most of the probable eggs were segmented. AS mentioned before the image was sectioned before processing to obtain images with a more homogeneous background, similar to the training images used by HEAD.

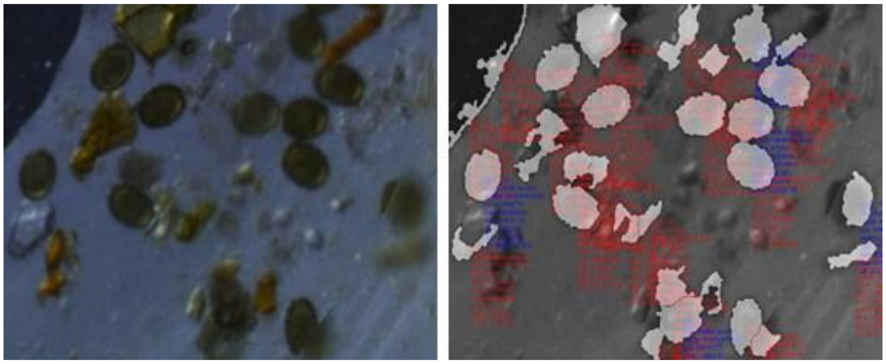

Image B. **Comparison of an original and processed FECPAKG2 image (detail).**

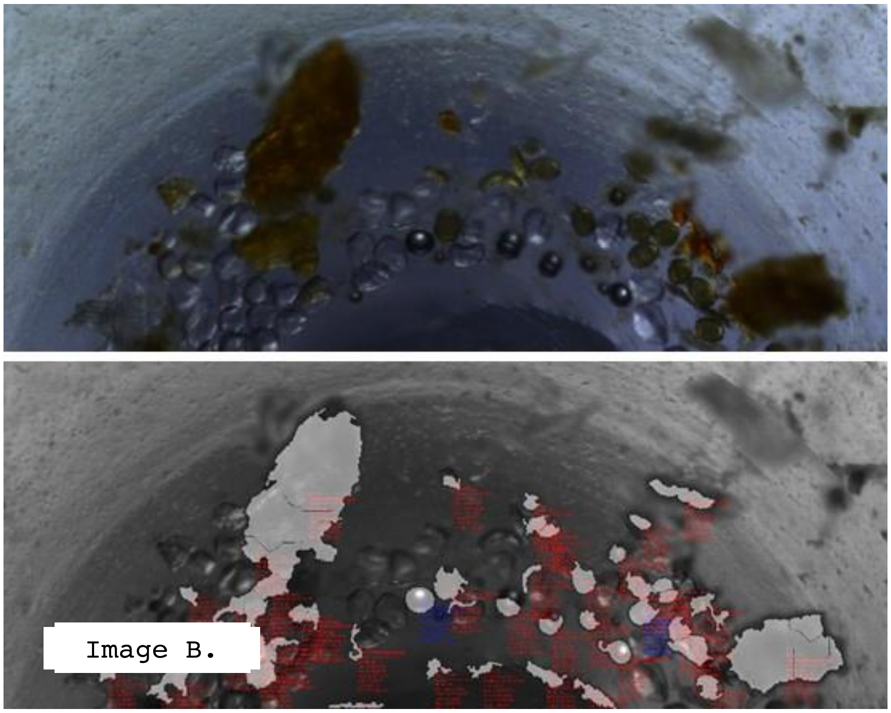

Image C **of an processed image**

Comparison original and FECPAKG2 (detail).

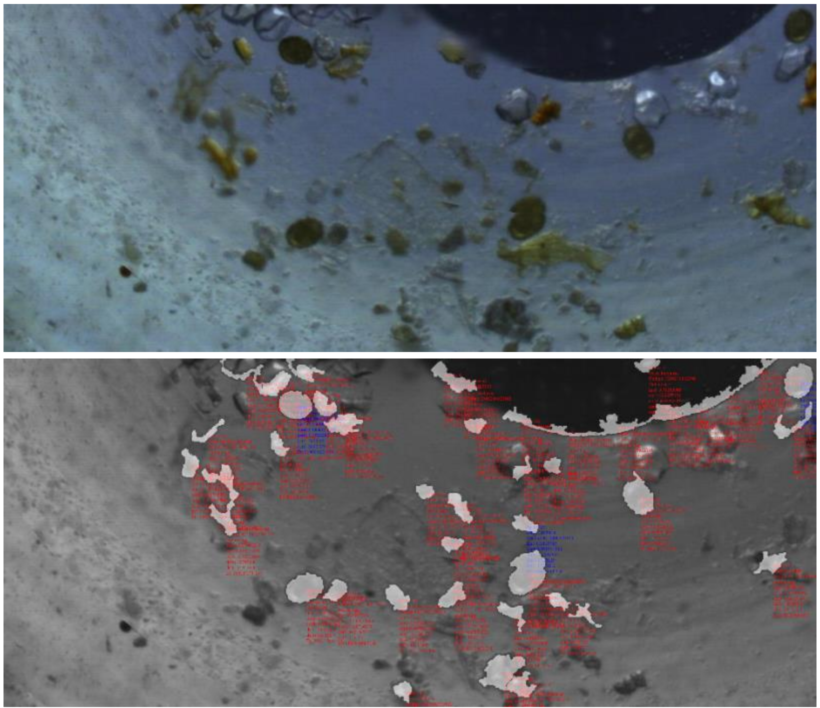

Image D. **Comparison of an original and processed FECPAKG2 image (detail).**

Classification

After HEAD analysis, most of the probable eggs were classified as "Not an egg" based on the statistical validation with HEAD's database, which indicated that their characteristics significantly differ from the metrics obtained for each helminth eggs species. Nonetheless, some of the well-focused eggs in image B were classified as **Fertile *Ascaris***. Table 1 shows a summary of the difference between some of the main characteristics of probable eggs and HEAD's database.

Table 1. Mean differences between FECPAKG2 objects and HEAD's database

Characteristic	Mean difference [%]
Morphology	5.33
Gray Level	30.77
Texture	21.26

From Table 1, it is clear that morphology was within the acceptable threshold for classification of helminth eggs by HEAD; however, gray level and texture characteristics were out of the acceptable ranges for adequate classification and thus objects were not identified as helminth eggs.

During HEAD analysis, it was observed that some of the relevant characteristics varied after scaling the image. According to UNAM estimations, the original FECPAKG2 image had to be resized about 250%, preserving objects' morphology but losing texture and gray level parameters due to a bidimensional interpolation process. This can be seen in image E where a fertile *Ascaris* egg from HEAD's database is compared to a probable *Ascaris* from the resized FECPAKG2 image.

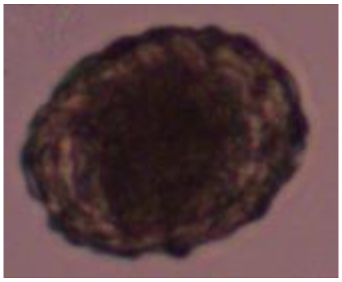
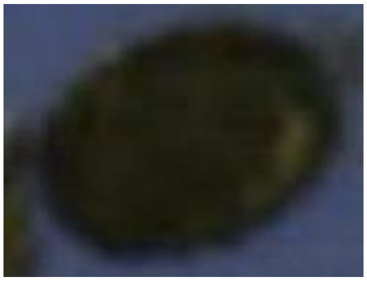

Image E. **Comparison between an egg from HEAD's database and probable egg from a scaled FECPAKG2 image.**

Table 2. Objects detected by HEAD

Objects	Number
Observed possible eggs (Original image)	25
Segmented possible eggs by HEAD	22

The percentage of probable Fertile *Ascaris* eggs segmented by HEAD in the 3 image sections software was 88%, but the classification and verification steps failed due to the texture and gray level values.

Preliminary conclusions

The scaled image submitted by the FECPAKG2 group could not be processed by HEAD due to its scale as well as the mean illumination of the background. As a result, it had to be divided into sub-images to be analyzed by HEAD. After processing different sections of the scaled FECPAKG2 image, most of the focused objects were correctly segmented. Some of those objects were even classified as "*Fertile* Ascaris". However, most of the probable eggs were not identified as such, due to the loss of information after resizing the image (interpolation). Based on these findings, it appears feasible to identify helminth eggs in FECPAKG2 images with HEAD, but further training and selection of parameters are needed to overcome variations in gray level and texture.
