# Supplementary material for: Diagnostic performance of a single and duplicate Kato-Katz, Mini-FLOTAC, FECPAKG2 and qPCR for the detection and quantification of soil-transmitted helminths in three endemic countries
Source: PLoS Negl Trop Dis. 2019 Aug 1;13(8):e0007446. doi: 10.1371/journal.pntd.0007446 (PMC6675048; doi:10.1371/journal.pntd.0007446)
Supplement: S6 Info — (PDF) [file pntd.0007446.s006.pdf]

S6 Info. Prevalence of helminths (other than *Ascaris*, *Trichuris* and hookworm) as determined by qPCR in the three study sites. For each parasite and study site, the number of cases on the number of samples analysed is shown, with the percentage in between brackets.

	**Ethiopia**	**Laos**	**Tanzania**
Schistosoma sp.	32/332 (9.6%)	2/478 (0.4%)	12/490 (2.5%)
Strongyloides stercoralis	10/332 (3.1%)	13/478 (2.8%)	23/490 (4.7%)
Taenia sp.	17/332 (5.0%)	14/478 (3.0%)	4/490 (0.8%)
Hymenolepis nana	14/100 (14.0%)	0/100 (0%)	0/130 (0%)
Enterobius vermicularis	10/100 (10.0%)	0/100 (0%)	0/130 (0%)
